# Zamilon, a Novel Virophage with *Mimiviridae* Host Specificity

**DOI:** 10.1371/journal.pone.0094923

**Published:** 2014-04-18

**Authors:** Morgan Gaia, Samia Benamar, Mondher Boughalmi, Isabelle Pagnier, Olivier Croce, Philippe Colson, Didier Raoult, Bernard La Scola

**Affiliations:** 1 Unité de Recherche sur les Maladies Infectieuses et Tropicales Emergentes, UMR CNRS-IRD 7278, IFR48, Faculté de Médecine, Aix-Marseille Université, Marseille, France; 2 Pôle des Maladies Infectieuses, Assistance Publique-Hôpitaux de Marseille, Marseille, France; University of Lausanne, Switzerland

## Abstract

Virophages, which are potentially important ecological regulators, have been discovered in association with members of the order *Megavirales*. Sputnik virophages target the *Mimiviridae*, Mavirus was identified with the *Cafeteria roenbergensis* virus, and virophage genomes reconstructed by metagenomic analyses may be associated with the *Phycodnaviridae*. Despite the fact that the Sputnik virophages were isolated with viruses belonging to group A of the *Mimiviridae*, they can grow in amoebae infected by *Mimiviridae* from groups A, B or C. In this study we describe Zamilon, the first virophage isolated with a member of group C of the *Mimiviridae* family. By co-culturing amoebae with purified Zamilon, we found that the virophage is able to multiply with members of groups B and C of the *Mimiviridae* family but not with viruses from group A. Zamilon has a 17,276 bp DNA genome that potentially encodes 20 genes. Most of these genes are closely related to genes from the Sputnik virophage, yet two are more related to *Megavirus chiliensis* genes, a group B *Mimiviridae*, and one to Moumouvirus monve transpoviron.

## Introduction

For over a decade, giant viruses of the nucleo-cytoplasmic large DNA virus (NCLDV) group have been extensively investigated [1]–[4]. This group is composed of the *Poxviridae*, which infect insects and vertebrates, the *Asfarviridae*, which infect swine, the *Iridoviridae*, which infect invertebrates and poikilothermic vertebrates, the closely related *Ascoviridae*, which infect insects, the *Phycodnaviridae*, which infect algae, and the putative *Marseilleviridae* family, which infect amoebae [5]. The *Acanthamoeba polyphaga Mimivirus* (APM) that was discovered in 2003 [6], as well as related viruses, cluster with the *Mimiviridae* family [7] within the NCLDV group. It has been proposed that the viruses of the NCLDV group should constitute a new viral order called *Megavirales* [8], [9]. The *Mimiviridae* family consists of 2 groups: the first group includes *Mimivirus*-like viruses that infect amoebae (Group I), while the second group is composed solely of the *Cafeteria roenbergensis* virus (CroV), which infects a marine heterotrophic bi-flagellate [10]. The *Mimiviridae* group currently consists of more than 40 *Mimivirus*-like viruses that infect the widespread amoeba genus *Acanthamoeba* and have been arranged in three lineages based on their pol B gene sequences [11], [12]. These lineages correspond to group A (which includes *Mimivirus* and Mamavirus), group B (Moumouvirus) [13], and group C (*Megavirus chiliensis*) [14]. The exploration of new giant viruses recently led to the identification of *Pandoraviruses* [15], which infect amoebae. *Pandoraviruses* form particles of approximately 1 µm that contain a 2.5 Mb-long DNA genome, in contrast to *Mimivirus*, which has 0.7 µm particles and a 1.2 Mb genome [16]. *Pandoraviruses* seem to cluster with the NCLDVs.

In 2008, a small virus with 50 nm icosahedral virions and an 18 kb genome was co-isolated with Mamavirus. This small virus infects the virus factory in which the giant virus replicates [17]. Due to its negative impact on the giant virus host, which is characterized by an increased production of abnormal particles and a decrease in infectivity and lytic ability, this small virus was dubbed the Sputnik virophage [18]. A second Sputnik strain, named Sputnik 2, was later isolated with the giant Lentillevirus, which, like Mamavirus, belongs to group A of the *Mimiviridae* [7], [19]. A third strain, Sputnik 3, was isolated with a *Mimivirus* reporter instead of with its natural viral host [11]. The three Sputnik virophages, which share more than 99% identity, have a broad host spectrum among the *Mimiviridae* and can replicate with viruses belonging to groups A, B and C [11]. In 2011, a new virophage was isolated in association with *Cafeteria roenbergensis* virus [20]. The detection from environmental datasets of abundant virophage genomes associated with *Mimiviridae*-related viruses or phytoplankton-infecting viruses led to the hypothesis that virophages play an important ecological role in the regulation of viral populations [21]–[25]. However, the nature of the virophages is still a matter of debate [26].

The aim of this study was to identify and characterize a new Sputnik-like virophage that was isolated previously [27]. This virophage was discovered in association with the Mont1 virus. Based on the Mont1 pol B gene sequence, this *Mimiviridae* family member belongs to group C. The virophage is thus the first described associated to this group. Amoebal cultures were co-infected with Zamilon and viruses from groups A, B and C to investigate the fitness of the virophage by transmission electron microscopy and real-time PCR. The virophage genome was then sequenced and analyzed.

## Material & Methods

### Isolation of Mont1 and Zamilon

The giant virus Mont1 and its virophage were isolated before this study from a soil sample collected in Tunisia using a high-throughput protocol [27]. Mont1 was identified a Mimivirus-like virus, with particles of approximately 500 nm in diameter surrounded by fibrils. The virus was classified as a group C *Mimiviridae* based on a partial sequence of the polymerase B gene (GenBank Accession No. JX484142). The virophage was Sputnik-like, i.e., had a spherical particle with a diameter of approximately 60 nm, and was named Zamilon (“the neighbor” in Arabic).

### Purification

To purify the giant virus, supernatants from amoebal co-cultures in PAS (Page's amoeba saline buffer) containing both Mont1 and Zamilon viruses were heat-inactivated. After 2 hours at 65°C, the absence of virophage particles was verified by negative staining electron microscopy, and the suspension was serially diluted up to 10^−10^ in PAS. End-point dilutions were performed in fresh *Acanthamoeba polyphaga* (strain Linc AP-1) cultures at a concentration of 5×10^5^ cells/mL. The most dilute sample that induced lysis was sub-cultured with fresh amoebae. The absence of virophage particles was verified again by negative staining electron microscopy, and the purified cultures containing only the giant virus Mont1 in *A. polyphaga* were stored at −80°C.

The Zamilon virophage was purified from a large volume (approximately 1.5 L) of supernatant from amoebae co-cultured with Mont1 and Zamilon in PYG. The supernatant was successively filtered through 0.8-, 0.45- and 0.22- µm membranes. The filtrate was then concentrated by ultracentrifugation at 25,000 rpm for 1.5 h with a SureSpin 630/36 rotor (Thermo Fischer Scientific, Waltham, MA, USA). The pellet was resuspended in 1 mL of PAS and then purified through a 15% sucrose layer by centrifuging at 25,000 rpm for 1.5 h. The highly concentrated pellet was resuspended in 1 mL of PAS and stored at −80°C after the absence of Mont1 particles was confirmed by negative staining electron microscopy. The Sputnik virophages were already purified with the same protocol and stored at −80°C from previous studies [7], [11], [17].

### Co-culture of the virophage

*A. polyphaga* was inoculated with the Zamilon virophage and several giant viruses that are representative of the 3 groups of *Mimiviridae* (APM and Mamavirus for group A, Moumouvirus and Monve virus for group B, and Courdo11 virus and Terra1 virus for group C). Zamilon was also co-cultured with its native host, Mont1 (group C *Mimiviridae*). The co-cultures were performed as previously described [11]. Briefly, 1 mL of filtered *Mimiviridae* and 100 µL of the purified virophage diluted 10-fold were added to 10 mL of fresh amoebae in PAS (5×10^5^ cells/mL). After 1 h of incubation at 32°C, the supernatant was removed, and the pellet was resuspended in 10 mL of PAS. The culture flasks were then incubated at 32°C. This time point was defined as H0. A 1 mL sample of each co-culture was removed at time point H16 (i.e., after 16 h of incubation) for transmission electron microscopy analysis, except for Mont1 and Mamavirus, for which samples were taken at H6, H8, H12, H16, H24 and H30. The number of amoebae was quantified at each time point using a KOVA Glasstic Slide (Hycor Biomedical Inc., Garden Grove, California, USA). The same protocol as described above was also performed with Courdo11 virus alone, with Sputnik or Zamilon, and samples were taken at H0 and H24.

### Plaque assay

Plaque assays were performed as previously described [11] with suspensions of Mont1 or Mamavirus with or without the Zamilon virophage. The virophage used in these experiments was diluted 10-fold from the frozen purified stocks. For each assay, 7.5 µL of the giant virus at a concentration of 10^9^ pfu/mL was combined with 7.5 µL of the diluted virophage or 7.5 µL of PAS and added to the plates. The plates were monitored daily for plaque formation, and diameters of the plaques were measured with calipers.

### Real-time PCR

DNA extractions and real-time PCR were performed using 200 µL of each co-culture taken at H0 and H16. Additional samples were taken from the co-cultures of Zamilon with Mont1 or Mamavirus at H6, H8, H12, H24 and H30, and from the co-cultures of Zamilon or Sputnik with Courdo11 virus at H0 and H24. The EZ1 DNA Tissue Kit (Qiagen, Hilden, Germany) was used for DNA extraction according to the manufacturer's instructions. A LightCycler 480 SYBR Green I Master (Roche Applied Science) was used to perform the real-time PCR according to the manufacturer's instructions. The following primers were used to detect the Zamilon virophage: forward primer 5′-GGGATGAACATCAAGCTGGT-3′ and reverse primer 5′-GGGTTGTTGGAAGCTGACAT-3′. The primers used for the quantification of Courdo11 virus were previously described [11].

### Sequencing and bioinformatics analysis

The Zamilon genome sequence was obtained using a MiSeq sequencer (Illumina), the MIRA assembler [28] and CLC Genomics Workbench version 4.9 (CLC BIO Aarhus, Denmark). Gene predictions were performed using GeneMarkS [29] and Prodigal [30] software with default parameters. The genome was annotated manually based on protein homology using BLASTp searches (Basic Local Alignment Search Tool) against the non-redundant protein collection in the NCBI database (http://http://blast.ncbi.nlm.nih.gov/Blast.cgi) and conserved domains were predicted using BLASTp, PSI-BLAST (Position-Specific Iterated BLAST) and InterProScan [31]. Nucleotide sequence comparisons were made using BLASTn searches against the nucleotide collection in the NCBI database. The genome architecture of the virophages and the *Mimiviridae* family members were compared using MUMmer [32]. Multiple sequence alignments were performed using MUSCLE [33] and curated using Gblocks [34]. Phylogenetic trees were constructed using the PhyML Maximum Likelihood algorithm [35]. The trees were visualized using MEGA v5 [36].

## Results

### Selective growth of the virophage in *Mimiviridae*

**Figure 1:**
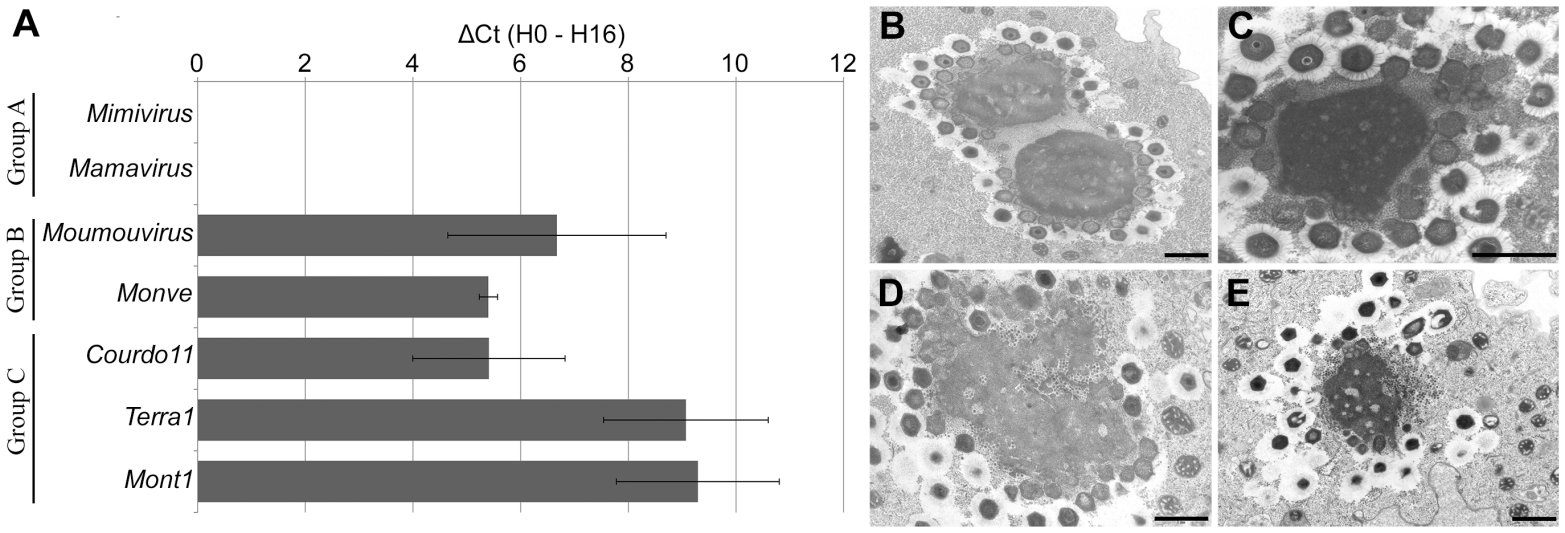
Zamilon growth in *Mimiviridae*. (**A**) Histogram of Zamilon growth in group A, B and C *Mimiviridae* family members, measured by real-time PCR. The difference in the Cycle threshold (Ct) between time points H0 and H16 is shown. (**B–E**) Transmission electron microscopy images of the virus factory in amoebae co-infected with Zamilon and *Mimiviridae*. No virophage particles were detected in the presence of Mimivirus (**B**; scale bar 0.1 µm), unlike Moumouvirus (**C**; scale bar 0.1 µm), Terra1 (**D**; scale bar 0.1 µm) and Mont1 (**E**; scale bar 0.1 µm).

Seven viruses from our laboratory's collection of the giant viruses were co-cultured with the Zamilon virophage: 2 viruses belonging to group A, 2 group B viruses, and 3 viruses from group C of the *Mimiviridae*. Based on real-time PCR and transmission electron microscopy analysis, Zamilon grew well with all of the viruses from group B and group C, but not with those belonging to group A of the *Mimiviridae* (Figure 1). Electron microscopy revealed that the virophage particles were spherical, with a diameter of approximately 50 to 60 nm. The particles appeared in the cytoplasm of the amoebae and were produced from the virus factory when the amoebae were co-infected with group B and group C *Mimiviridae*. Relative quantification by real-time PCR showed that the rate of virophage multiplication depends on the giant virus.

### The Zamilon genome

**Figure 2:**
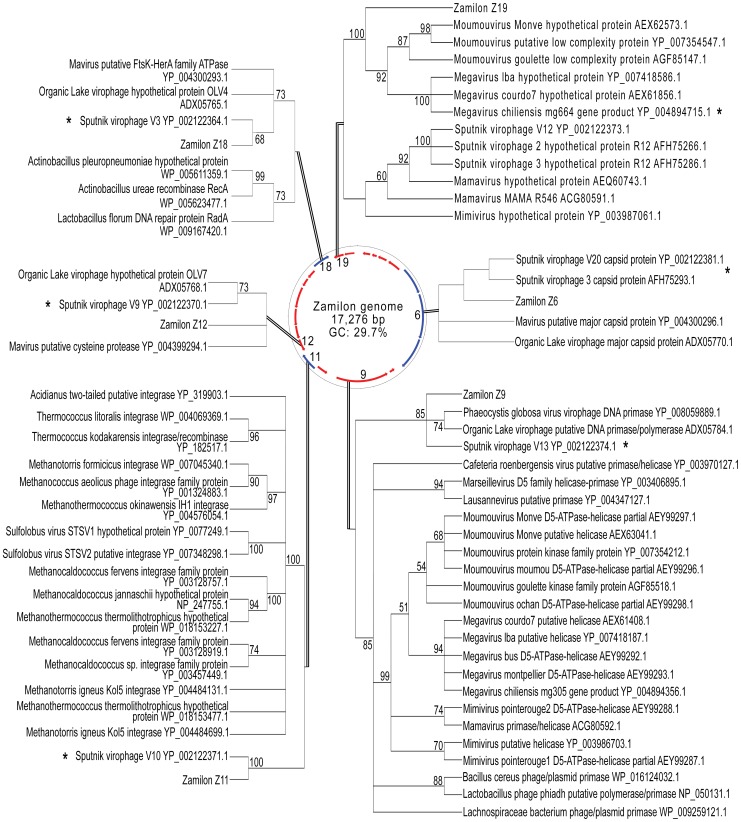
The Zamilon genome. The Zamilon genome, with predicted coding sequences on the forward strand (blue) and the reverse strand (red). Phylogenetic analyses of ORF6, 9, 11, 12, 18 and 19 are included with bootstrap values indicated (cutoff ≥50). * indicates the best hit (*E*-values: 0, 0, 8^−80^, 2^−90^, 5^−147^, and 5^−34^, respectively).

We sequenced the 17,276 bp circular genome of the Zamilon virophage (EMBL-EBI ID: HG531932.1). The GC content was 29.7%. Analysis of the whole genome at the nucleotide level showed that it is most similar to the Sputnik virophage (76% identity, 75% coverage). A total of 20 ORFs (Open Reading Frame) were identified by gene prediction and ranged from 222 bp to 2337 bp in length (Figure 2). Most of these ORFs had significant homology to predicted Sputnik virophage protein sequences (Table 1). However, a genomic dot plot of Zamilon and the Sputnik virophage showed that Zamilon constitutes a new virophage and that its genome contains a reversed portion from approximately 6,000 bp to the end (Figure S1). Reversed nucleotide sequences are also evident in the genome of a group A *Mimiviridae* family member (*Mimivirus*) compared to the genomes of viruses from groups B and C (Moumouvirus and *Megavirus chiliensis*, respectively) (Figure S1).

**Table 1:** Closest homologs of the Zamilon open reading frames (ORFs).

ORF (size in amino acids)	Closest homolog in GenBank nr (accession no.)	Identity/alignment length	*E*-value	Predicted function
ORF1 (111)*	Sputnik virophageV15 (YP_002122376.1)*	32%/97	0.081*	hypothetical protein*
ORF2 (73)*	Sputnik virophage V2 (YP_002122363.1)*	31%/62	7e-6*	hypothetical protein*
ORF3 (135)	Megavirus chiliensis mg3 gene product (YP_004894054.1)	67%/52	9e-14	hypothetical protein
ORF4 (221)	Sputnik virophage 2 putative IS3 family transposase A protein (AFH75271.1)	40%/48	0.003	putative transposase
ORF5 (376)	Sputnik virophage 2 minor virion protein (3J26_N)	66%/375	2e-178	minor virion protein
ORF6 (609)	Sputnik virophage putative capsid protein V20 (YP_002122381.1)	86%/609	0	capsid protein
ORF7 (442)	Sputnik virophage V21 (YP_002122382.1)	70%/442	0	hypothetical protein
ORF8 (81)	Moumouvirus Monve hypothetical protein tv_L8 (AEY99266.1)	72%/53	4e-18	hypothetical protein
ORF9 (778)	Sputnik virophage V13 (YP_002122374.1)	67%/778	0	putative helicase
ORF10 (168)	Sputnik virophage V11 (YP_002122372.1)	53%/165	6e-44	hypothetical protein
ORF11 (247)	Sputnik virophage V10 (YP_002122371.1)	58%/217	8e-80	putative integrase
ORF12 (175)	Sputnik virophage V9 (YP_002122370.1)	77%/175	2e-90	hypothetical protein
ORF13 (184)	Sputnik virophage V8 (YP_002122369.1)	71%/184	5e-82	structural protein
ORF14 (241)	Sputnik virophage V7 (YP_002122368.1)	80%/241	3e-120	hypothetical protein
ORF15 (305)	Sputnik virophage V6 (YP_002122367.1)	75%/314	9e-136	collagen-like protein
ORF16 (121)	Sputnik virophage V5 (YP_002122366.1)	59%/86	1e-31	hypothetical protein
ORF17 (133)	Sputnik virophage V4 (YP_002122365.1)	55%/143	5e-44	hypothetical protein
ORF18 (245)	Sputnik virophage V3 (YP_002122364.1)	81%/245	5e-147	DNA packaging - ATPase
ORF19 (147)	Megavirus chiliensis mg664 gene product (YP_004894715.1)	50%/129	5e-34	hypothetical protein
ORF20 (147)	Sputnik virophage V1 (YP_002122362.1)	60%/126	2e-18	hypothetical protein

Best hit for Zamilon's ORFs obtained with BlastP against the non-redundant (nr) NCBI database.

Hypothetical functions were determined by homology and conservation of protein domains. * indicates ORFs with no significant homology in the nr database. These ORFs were aligned directly to the Sputnik virophage.

Fifteen different ORFs (ORF4-ORF7, ORF9-ORF18 and ORF20) showed between 40 and 80% homology in amino acids to predicted genes from the Sputnik virophage. Some of the predicted proteins have functions, including a putative transposase, capsid-forming proteins, a collagen-like protein, a helicase, an integrase and an ATPase (Figure S2). The closest homolog to ORF12 was the Sputnik V9 gene, which encodes an unidentified protein. However, BLAST alignments showed that this ORF was also related to a putative cysteine protease protein from the Mavirus virophage (32% identity and 83%coverage, *E*-value 4^−17^; GenBank Accession No. YP_004300284.1). Similarly, ORF17 is related to the uncharacterized Sputnik V4 gene and also to a zinc-finger C2H2-type domain-containing protein. Phylogenetic analysis of ORF11 and ORF18 confirmed that they are closely related to Sputnik genes that potentially encode an integrase and a DNA-packaging protein with a putative ATPase domain, respectively (Figure 2). The predicted protein sequence encoded by ORF9, which encodes a putative helicase, also shows homology to the *Organic Lake Virophage* putative DNA primase/polymerase (GenBank Accession No. ADX05784.1) and to the putative DNA primase from the virophage associated with *Phaeocystis globosa* (GenBank Accession No. YP_008059889.1) (Figure 2).

The Zamilon ORF3 shows significant homology to the *Megavirus chiliensis* mg3 gene. Annotation of ORF19 showed homology to the *Megavirus chiliensis* mg664 gene, and this homology was further confirmed by phylogenetic analysis (Figure 2). This ORF clustered closer to the group B and C *Mimiviridae* viruses than to the group A *Mimiviridae* viruses and their associated Sputnik virophages. Significant homology was detected between the Zamilon ORF8 protein sequence and the hypothetical protein tv_L8 from the transpoviron Moumouvirus monve, which does not have a predicted function. Transpovirons are linear mobile DNA elements dependent of the giant virus infection and able to integrate the *Mimiviridae* and the virophage genomes [19]. The Zamilon ORF8 is also homolog to Sputnik V14 gene, which does not have any predicted function.

ORF1 and ORF2 did not exhibit significant similarity or homology to any entries in the NCBI databases and were therefore aligned with the Sputnik virophage genome (GenBank Accession No. NC_011132.1). Zamilon ORF1 and ORF2 showed some homology to the Sputnik V15 and V2 genes, respectively (≥30% identity; *E*-values 0.081 and 7^−06^, respectively). The predicted protein sequence encoded by ORF1 contained a putative conserved protein domain related to a transmembrane domain from cytochrome c oxydase subunit II (*E*-value 2.3e-4; EMBL-EBI ID: IPR011759).

### The impact of the Zamilon virophage on its host

**Figure 3:**
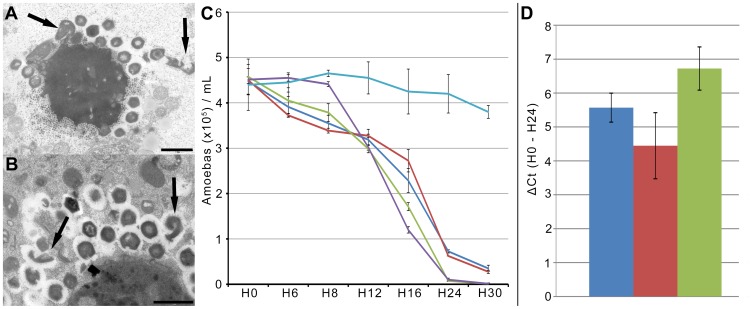
Impact of the Zamilon virophage. (**A–B**) Transmission electron microscopy images of abnormal Mont1 virus particles (arrows) produced from the virus factory with (**A**; scale bar 0.1 µm) and without (**B**; scale bar 0.1 µm) Zamilon. (**C**) Kinetics of survival of amoebae infected with Mont1 or Mamavirus, with or without the Zamilon virophage (Blue: Mont1, Red: Mont1 and Zamilon, Green: Mamavirus, Purple: Mamavirus and Zamilon, Turquoise: negative control). The x-axis shows the time points, and the y-axis shows the number of amoebae per milliliter (×10^5^ cells/mL). (**D**) Histogram of Courdo11 virus growth alone (blue), with Sputnik (red) and with Zamilon (green). The difference in the Cycle threshold (Ct) between time points H0 and H24 is shown.

There was no significant difference in the diameters of the plaques formed by the giant viruses Mont1 and Mamavirus whether they were co-inoculated with or without the Zamilon virophage. However, Mont1 formed sun-like plaques, while Mamavirus formed rounded plaques (Figure S3). Transmission electron microscopy revealed a high proportion of abnormal Mont1 particles when the virus was co-cultured with the Zamilon virophage (Figure 3A). However, a similar number of abnormal particles were observed when Mont1 was cultured alone (Figure 3B). When the Zamilon virophage was co-cultured with other members of *Mimiviridae* groups B and C, the proportion of abnormal giant particles produced remained unchanged. Moreover, co-culture with Zamilon had no effect the ability of Mont1 or Mamavirus to induce lysis in the amoebal host (Figure 3C), and quantification of the multiplication of Courdo11 virus, a group C *Mimiviridae*, with Sputnik or Zamilon in 24 hours showed a higher multiplication of the giant virus with Zamilon than with Sputnik (Figure 3D).

## Discussion

In this study, we report the identification and characterization of a novel virophage, Zamilon, that is associated with giant viruses from the *Mimiviridae* family. This virophage is closely related to the Sputnik virophages and is the first virophage isolated together with a member of *Mimiviridae* group C.

The Zamilon virophage particles are spherical, with a diameter of 50 to 60 nm, similar to Sputnik and Mavirus particles [17], [20], [21]. The Zamilon genome is also similar in size to known virophage genomes, as it is approximately 17 kb and contains 20 putative ORFs. In comparison, the Sputnik genome is 18 kb long and contains 20 ORFs, and the Mavirus genome is 19 kb long and encodes 20 ORFs [17], [20]. The 19 kb genome of the *Phaeocystis globosa* virus virophage encodes 16 proteins [24], and virophage genomes detected by metagenomic analysis of environmental samples exhibit similar characteristics. The Organic Lake virophage has a 26 kb genome that contains 26 predicted genes [21], [24], and other virophage genomes constructed from metagenomic datasets range from 17 to 27 kb and contain 21 to 26 ORFs [22].

Several Zamilon virophage ORFs encode proteins that share sequence homology or conserved domains with predicted proteins from several virophages, such as ATPase, helicase, integrase, transposase and capsid proteins [11], [17], [20], [21] (Figure S2). These genes represent a set of core functions for these viruses [37]. In addition, most of the predicted Zamilon virophage proteins exhibited moderate to high homology to predicted Sputnik virophage proteins, as well as proteins encoded by *Megavirus chiliensis* and the Moumouvirus monve transpoviron.

In contrast to the Sputnik virophages [11], Zamilon does not seem to have a significant impact on the giant virus host. Indeed, the Sputnik virophage increases the rate of abnormal giant virus particles and decreases the lytic and infective capacity of the giant virus [17]. Zamilon did not affect the rate of abnormal particle formation, nor did it affect the ability of the giant virus to lyse infected amoebae. More, it did not seem to reduce the multiplication of the giant virus at 24 h p.i., contrarily to Sputnik. These data suggest the absence of impact of Zamilon on giant viruses, even if further studies are required to fully understand this feature on all the *Mimiviridae* collection. If confirmed, it would question the concept of virophage. Indeed, the strongest argument in favor of this concept relies on the negative effect the virophage has on its host virus. It is possible that this group is basically composed of viruses with some members, like Sputnik, that have acquired the ability to reduce their host virus fitness, or that viruses derived from virophages with a particular adaptation to their host have lost this ability. Till now, only Sputnik virophages and Zamilon have been tested with several *Mimiviridae* viruses, and it would require more virophages' isolations and studies to really appreciate their true nature.

The three Sputnik virophages that were previously described have a broad host spectrum and can replicate with *Mimiviridae* from groups A, B and C [11]. The Sputnik virophage and Sputnik 2 were isolated with Mamavirus and Lentillevirus, respectively, both of which are group A *Mimiviridae* [7], [17]. Sputnik 3, however, was isolated alone, without a giant virus, and was presumed to be associated with a member of the group C *Mimiviridae* [11]. Despite these differences, the 3 Sputnik strains share more than 99% nucleotide similarity. In contrast, the Zamilon virophage was isolated with Mont1, a group C *Mimiviridae*, and is thus the first virophage known to be associated with this group [27]. This novel virophage is unable to grow in association with group A *Mimiviridae* (as assessed by transmission electron microscopy and real-time PCR), despite its similarity to *Sputnik* virophages. The Zamilon virophage contains a predicted gene that is more related to *Megavirus chiliensis*, a group C *Mimiviridae*, than to Sputnik virophages, and a predicted gene that has a homology only in *Megavirus chiliensis*. These genes could partly explain this novel host specificity. In particular, Zamilon ORF19 appears to be closely related to *Megavirus chiliensis* mg664. Phylogenetic analysis of this ORF showed that it clusters closer to members of *Mimiviridae* group B and C than to the Sputnik virophages associated with group A. We did not identify a putative function or any conserved protein domains for this ORF. Thus, it is difficult to evaluate the potential role that this gene plays in host virus genotype specificity, although we speculate that it is likely to be a factor in this selectivity. This host selectivity has also been described in bacteriophages, as some bacteriophages are specific to a single bacterial species within a microbial community or even only a few strains within a single species [38]–[40]. Changes in bacteriophages host ranges could arise due to nucleotide or protein mutations [41], [42]. These mutations could induce changes in the balance between phage-infectivity and host-resistance, which could lead to host specificity [43], [44]. Infectivity requires several steps that are shared by all viruses, including virophages, from recognition of the host, to entry and transport to the replication compartment, to replication itself. We hypothesize that the Zamilon virophage ORF19, which clusters with the group B and C *Mimiviridae*, plays a role in one of these stages of infection, along with other factors and maybe other ORFs, such as the Zamilon ORF3 which as only a homolog in *Megavirus chiliensis*.

The mechanism and timing of viral host selection remains unknown. The Sputnik virophages presumably take advantage of the phagocytosis of their giant virus hosts to enter the amoebal host [45]. Indeed, amoebae from the *Acanthamoeba* genus can internalize even particles greater than 0.5 µm in diameter, including latex beads, and it has been hypothesized that the Sputnik virophages penetrate the amoeba by attaching themselves to *Mimiviridae* fibrils during phagocytosis [45], [46]. Structural studies have revealed fibers protruding from the surface of Sputnik that do not have a clear function and may be associated with this host virus recognition [47], [48]. The host virus genotype specificity exhibited by Zamilon may involve recognition of a specific pattern on the surface of the giant virus. Once internalized, the Sputnik virophages multiply in the viral factory formed by the associated giant viruses [17], [20], [26]. However, as the interactions between functional *Mimiviridae* proteins and the virophages during replication are not clearly identified, specific sequence recognition cannot be ruled out.

Virophages are suspected to be key players in the regulation of environmental virus populations [21]. They may reduce the infectivity, and thus the reproductive fitness, of viruses, thus decreasing host mortality [22], [23], [25]. This regulation of global ecology through virus-induced cell lysis suggests that more virophage lineages remain to be discovered that target viruses implicated in environmental ecologies. Our results show that, even within a single lineage, virophages are more complex than initially thought and can target specific genotypes within in a virus family.

The host-specificity of the Zamilon virophage supports the distinction between satellite viruses (opportunistic entities associated with a virus) and virophages, which target specific hosts.

## Supporting Information

Figure S1 **Comparisons of virophages and** ***Mimiviridae*** **genomes.** (**A**) Comparison of the Zamilon genome to the Sputnik genome. (**B–D**) Comparisons of *Mimiviridae* genomes depending on the group they belong to: group A Mimivirus compared to group B Moumouvirus (**B**), group A Mimivirus compared to group C *Megavirus chiliensis* (**C**), and group B Moumouvirus compared to group C *Megavirus chiliensis* (**D**).
(TIF)

Figure S2 **Putative functions in virophages.** Genes encoding hypothetical and putative functions shared among the Zamilon, Sputnik, Mavirus, Phaeocystis globosa virus (PgVV) and Organic Lake (OLV) virophages are shown in the same color. Function predictions were made according to homologies between virophages or to nr NCBI collection, or regarding conservation of protein domains.
(TIF)

Figure S3 **Lysis plaque assay with Mont1 and Mama.** Scan of colored lysis plaques with *A. polyphaga* monolayer inoculated with Mont1 (**A**) and Mamavirus (**B**) 3 days after inoculation. Magnification of a Mont1 spot (**C**).
(TIF)
