# Supplementary figures and images for: Zamilon, a Novel Virophage with Mimiviridae Host Specificity
Source: PLoS One. 2014 Apr 18;9(4):e94923. doi: 10.1371/journal.pone.0094923 (PMC3991649; doi:10.1371/journal.pone.0094923)

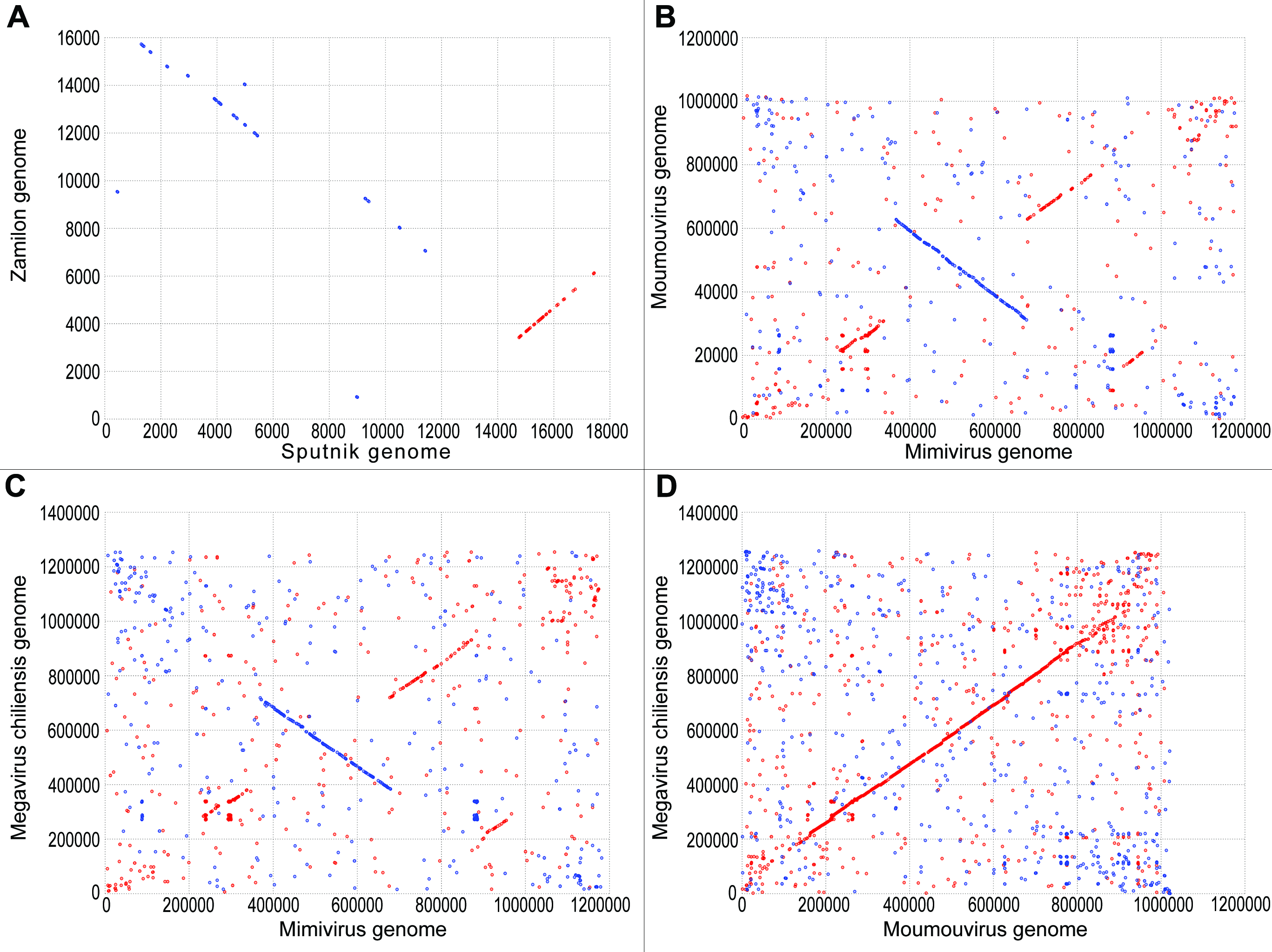

Supplement: Figure S1 — Comparisons of virophages and Mimiviridae genomes. (A) Comparison of the Zamilon genome to the Sputnik genome. (B–D) Comparisons of Mimiviridae genomes depending on the group they belong to: group A Mimivirus compared to group B Moumouvirus (B), group A Mimivirus compared to group C Megavirus chiliensis (C), and group B Moumouvirus compared to group C Megavirus chiliensis (D). (TIF) [file pone.0094923.s001.tif]

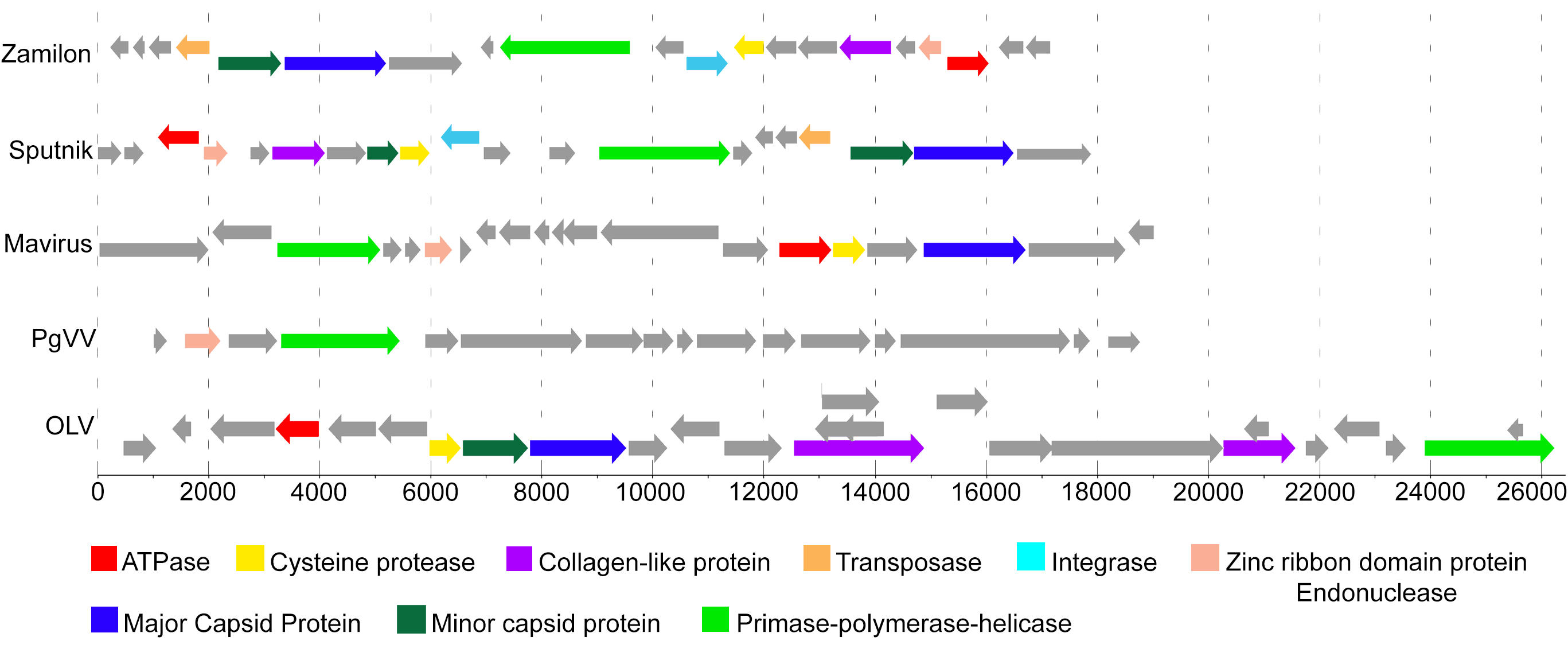

Supplement: Figure S2 — Putative functions in virophages. Genes encoding hypothetical and putative functions shared among the Zamilon, Sputnik, Mavirus, Phaeocystis globosa virus (PgVV) and Organic Lake (OLV) virophages are shown in the same color. Function predictions were made according to homologies between virophages or to nr NCBI collection, or regarding conservation of protein domains. (TIF) [file pone.0094923.s002.tif]

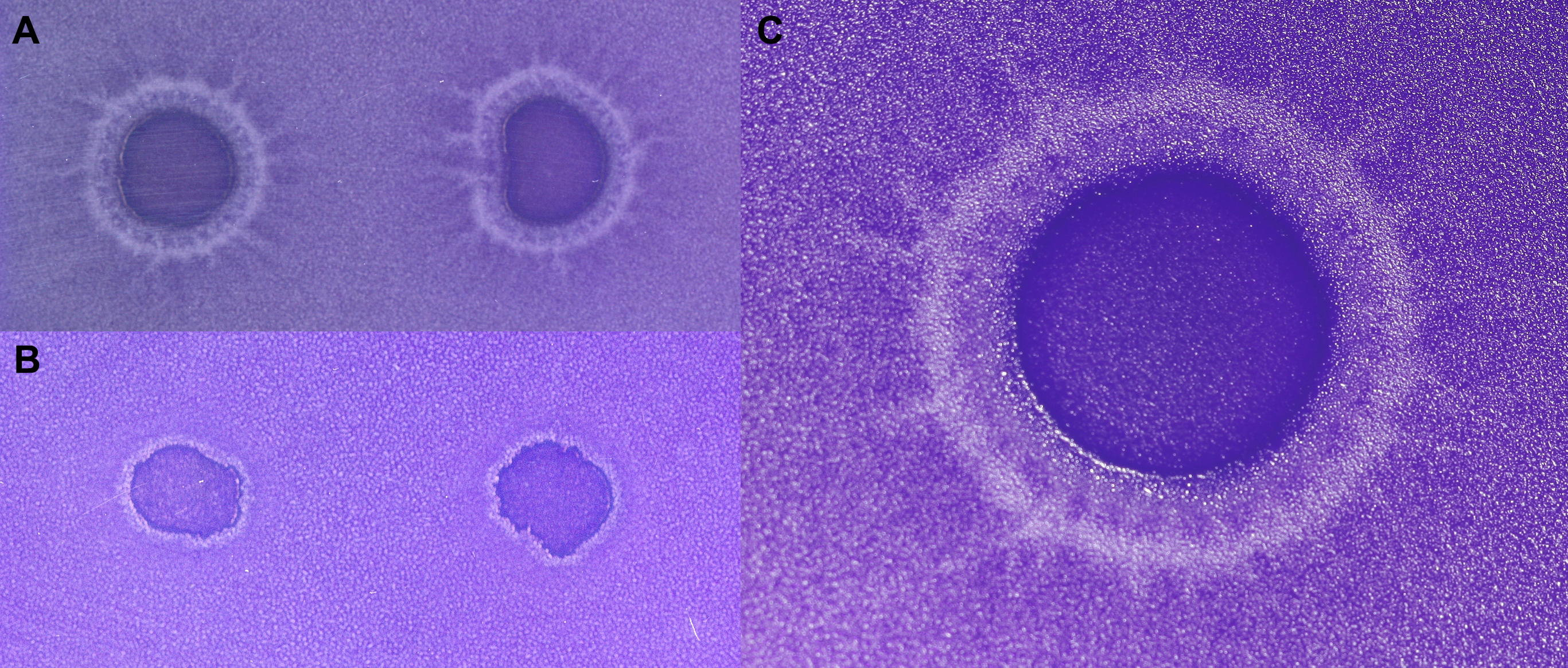

Supplement: Figure S3 — Lysis plaque assay with Mont1 and Mama. Scan of colored lysis plaques with A. polyphaga monolayer inoculated with Mont1 (A) and Mamavirus (B) 3 days after inoculation. Magnification of a Mont1 spot (C). (TIF) [file pone.0094923.s003.tif]
